# Bioengineering of a Full-Thickness Skin Equivalent in a 96-Well Insert Format for Substance Permeation Studies and Organ-On-A-Chip Applications

**DOI:** 10.3390/bioengineering5020043

**Published:** 2018-06-07

**Authors:** Katharina Schimek, Hao-Hsiang Hsu, Moritz Boehme, Jacob Jan Kornet, Uwe Marx, Roland Lauster, Ralf Pörtner, Gerd Lindner

**Affiliations:** 1 TissUse GmbH, D-13347 Berlin, Germany; katharina.schimek@tissuse.com (K.S.); uwe.marx@tissuse.com (U.M.); 2 Institute of Biotechnology, Department Medical Biotechnology, Technische Universität Berlin, D-13355 Berlin, Germany; roland.lauster@tu-berlin.de (R.L.); gerd.lindner@tu-berlin.de (G.L.); 3 Institute of Bioprocess and Biosystem Engineering, Hamburg University of Technology, D-21073 Hamburg, Germany; hao.hsu@tuhh.de (H.-H.H.); moritz.boehme@tuhh.de (M.B.); jacob.kornet@tuhh.de (J.J.K.)

**Keywords:** full thickness skin equivalents, multi-organ chip, substance permeation, 96-well cell culture insert

## Abstract

The human skin is involved in protecting the inner body from constant exposure to outer environmental stimuli. There is an evident need to screen for toxicity and the efficacy of drugs and cosmetics applied to the skin. To date, animal studies are still the standard method for substance testing, although they are currently controversially discussed Therefore, the multi-organ chip is an attractive alternative to replace animal testing. The two-organ chip is designed to hold 96-well cell culture inserts (CCIs). Small-sized skin equivalents are needed for this. In this study, full-thickness skin equivalents (ftSEs) were generated successfully inside 96-well CCIs. These skin equivalents developed with in vivo-like histological architecture, with normal differentiation marker expressions and proliferation rates. The 96-well CCI-based ftSEs were successfully integrated into the two-organ chip. The permeation of fluorescein sodium salt through the ftSEs was monitored during the culture. The results show a decreasing value for the permeation over time, which seems a promising method to track the development of the ftSEs. Additionally, the permeation was implemented in a computational fluid dynamics simulation, as a tool to predict results in long-term experiments. The advantage of these ftSEs is the reduced need for cells and substances, which makes them more suitable for high throughput assays.

## 1. Introduction

The skin, the largest human organ, offers important functions. It protects the body against mechanical influences, ultraviolet light, temperature, and dehydration. In addition, the skin has sensor functions responding to touch, temperature, and pain. Apart from other biochemical and physiological functions, the skin serves as a physical barrier between the human body and the surrounding environment [1]. Understanding this barrier’s function and its reactions to topically applied substances is fundamental for cosmetic and pharmacological applications. Consequently, the need to screen for the toxicity and efficacy of the skin is evident. Historically, a huge number of animal trials have been performed worldwide to address this purpose. Tests on skin irritation, for example, have been carried out using the Draize skin irritation test in rabbits, according to OECD TG 404 (Organization for Economic Co-operation and Development) [2]. However, there are several differences between animal and human data, for example, leading to the misclassification of chemicals using in-vivo rabbit data [3,4]. In addition, the EU Cosmetics Regulation No. 1223/2009 came into law in 2013, and prohibits the testing of cosmetic products on animals. This increases the demand for alternative methods [5,6]. Nowadays, human skin equivalents are widely used for animal-free tests in drug development, the chemical and cosmetics industries, and for research purposes. There are currently several skin equivalents commercially available: for example, EpiSkin™ (L’oreal, Clichy, France); EpiDerm™ SIT (MatTek Corporation, Ashland, MA, USA); SkinEthik™ RHE (SkinEthic laboratories, Lyon, France); EpiCS^®^ (CellSystems, Troisdorf, Germany); and LabCyte EPI-MODELS24 SIT (Japan Tissue Engineering Co., Tokyo, Japan). These models are all based on only one cell type mimicking the epidermis. However, full-thickness skin equivalents (ftSEs) with an additional dermal layer are also available on the market (e.g., GraftSkin^®^, EpiDermFT^®^, and Phenion^®^). These models are already commonly used for substance absorption tests, in order to understand the toxicity and efficacy of pharmaceutical and cosmetic products applied to the skin [5,7,8]. The Franz diffusion cell system is a method very widely used to determine the absorption or permeation of a substance into the skin [9,10].

A further trend for alternatives to animal substance testing, which does not focus solely on the skin, is the organ-on-a-chip system. The basic idea behind this approach is to emulate the smallest physiological unit of an organ on a chip. Thus, microenvironments must be created that correspond to the organs’ natural space. The influence of substrate topography, fluidic shear stress, and culture perfusion are important factors in organ cultivation in the chip format [11,12]. Reproducing diseases on the chip to test the effectiveness of active substances is of great interest to the pharmaceutical industry. Therefore, long-term cultivation also plays an important role [13]. The variety of organ-on-a-chip-based systems is enormous. Various microfluidic perfusion culture systems have been developed on a research basis, and commercialization of these platforms keeps growing (e.g., μOrgano, ChipShop, Emulate, TissUse, Nortis, AxoSim, or Draper).

A few groups have reported skin-related cultures in chip systems [14,15,16,17]. However, looking at commercially available skin models and organ-on-a-chip systems, it becomes apparent that there is no ftSE produced in a 96-well cell culture insert (CCI) format. We have recently described a 96-well CCI-based culture within the two-organ chip (2OC) of either punched skin equivalents, commercially available in a 24-well CCI format, or native skin biopsies [18,19,20]. This article presents a new approach to producing ftSEs in a 96-well CCI size, which is suitable for organ-on-a-chip systems. The ftSE consists of primary human skin cells within a collagen–elastin matrix (Matriderm^®^). This matrix is provided as cellular lyophilized sheets composed of bovine elastin and collagen types I, III, and V. It is used most frequently in clinics as a tissue replacement, supporting the ingrowth of cells and vessels for proper regeneration of injured skin [21].

In an already published study, a method was investigated to determine and simulate the permeation of substances in a 96-well CCI system [22,23]. Permeation is a helpful parameter that can describe the barrier properties of the skin. The diffusion coefficient can be determined with the help of a simulation, using a numerical optimization method. Here, these methods are modified for use in an organ-on-a-chip system with ftSEs for future applications. In this study, the 2OC developed by TissUse GmbH was used [13,19,24,25,26,27,28]. This system, about the area of a microscope slide, enables the cultivation of different organ types on a single chip. It consists of a polycarbonate adapter plate supporting the connectors for 96-well Transwell^®^ holders and the pump. Two culture compartments in one fluid circulation unit enable cultivation of two different tissue types. The fluid channel is made of polydimethylsiloxane (PDMS), and connects the two culture compartments supplying the organ models with nutrient solution. A micro-pump system, inspired by the human cardiovascular system [29], is operated with compressed air and enables the fluid flow. The 2OC links metabolic interaction of different organ types, thereby mimicking a part of the human body. This approach is well-established for the co-culture of human liver and skin tissue [19,20,26,30]. One promising aspect for the future application of the 2OC is dermal in-vitro substance testing, in combination with other tissues (e.g., liver, kidney, heart).

## 2. Materials and Methods

### 2.1. Cell Isolation and Culture

Normal human keratinocytes (NHKs) and human dermal fibroblasts (HDFs) were isolated from prepuce biopsies (EA2/091/12 of the Ethic Committee Charité University Medicine and Eth-10/15 of the Berlin Chamber of Physicians, Berlin, Germany). The prepuce was cleaned in 80% ethanol for 30 s and rinsed with phosphate-buffered saline (PBS) prior to isolation. The subcutaneous tissue was removed and incubated in 5 mg/mL dispase II solution (Sigma-Aldrich, St. Louis, MO, USA) at 4 °C for 15–18 h to separate the thin epidermal layer from the dermis.

For the isolation of NHK, the epidermis of the biopsy was cut into small pieces and incubated in trypsin/ethylenediaminetetracetic acid (EDTA) (Corning, Amsterdam, The Netherlands) for 15 min at 37 °C. The suspension was then passed through a 70 µm nylon filter (BD Falcon™ Cell Strainer, Erembodegem, Belgium) and centrifuged for 5 min at 300× *g*. The supernatant was aspirated and the pellet re-suspended in 1 mL EpiLife medium + 1% human keratinocyte growth supplement (HKGS) (Gibco/Life Technologies, Darmstadt, Germany), with 1% Penicillin/Streptomycin (Corning, Amsterdam, The Netherlands) (E1 medium). Cells were seeded at a density of 1.3 × 10^4^ cells/cm^2^ into collagen I-coated (Biochrom GmbH, Berlin, Germany) flasks with E1 medium. The medium was changed twice a week. After a few days of culture, when cells reached 80% confluence, the NHKs and melanocytes were separated by differential trypsination. Trypsin/EDTA was added in the flask and incubated for 4–5 min at 37 °C to remove the melanocytes. In a second trypsination step, with an incubation time of 10–15 min, the NHKs were passaged into new collagen-coated flasks. The NHKs were then harvested at 80% confluency, frozen in fetal calf serum (FCS) (Corning, Amsterdam, The Netherlands) with 10% dimethyl sulfoxide (DMSO) (Carl Roth, Karlsruhe, Germany), and stored in liquid nitrogen until use.

For the isolation of HDFs, the dermis of the prepuce biopsy was cut into strips and incubated for 45 min in Dulbeccos’s Modified Eagle Medium (DMEM) (Corning, Amsterdam, The Netherlands), containing 4 mg/mL collagenase NB 4 (Serva Electrophoresis GmbH, Heidelberg, Germany). The suspension was then passed through a 70 µm nylon filter, centrifuged for 5 min at 300× *g*, and the pellet was re-suspended in a 1 mL medium composed of DMEM, including glutamine, high glucose (Corning, Amsterdam, The Netherlands) with 1% Penicillin/Streptomycin, and 10% FCS (D10). Cells were seeded at 2 × 10^4^ cells/cm^2^ and passaged once after reaching 80% confluence. The medium was changed one day after seeding, and then every three to four days. When the HDF reached 80% confluence again, the cells were harvested, frozen in FCS with 10% DMSO, and stored in liquid nitrogen until used.

### 2.2. Manufacturing of Full Thickness Skin Equivalents

**Figure 1:**
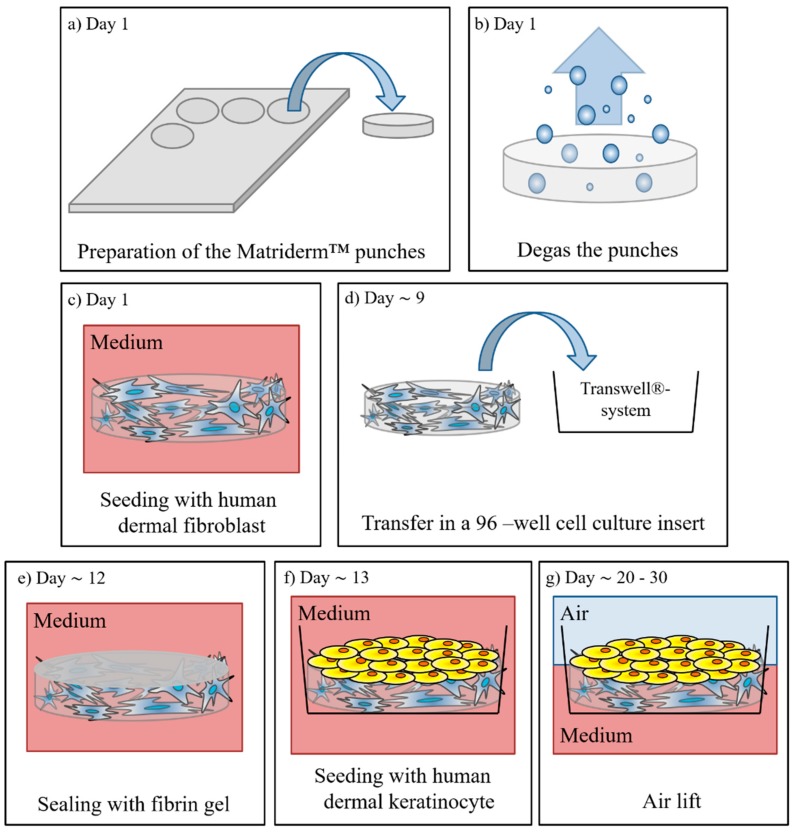
Schematic diagram of the ftSE cultivation. (**a**) Punches are cut out of the Matriderm™ matrix and (**b**) degassed in an exsiccator. (**c**) Then the matrix is seeded with human dermal fibroblasts. (**d**) On day nine, the ftSE is transferred into a cell culture insert system. (**e**) After four days of cultivation, the skin equivalent is sealed with a fibrin gel. (**f**) One day later, normal human keratinocytes are seeded onto the top of the equivalent. (**g**) On day 20, the equivalents are lifted to the air-liquid interface and cultivated further for ~10 days.

1 mm thick Matriderm^®^ (asclepiosMedizintechnik, Gutach, Germany) pieces 4.5 mm in diameter were punched out and degassed in PBS (Biochrom GmbH, Berlin, Germarny) within an exsiccator (Nalgene^®^, Rochester, NY, USA) (see Figure 1a,b). The Matriderm^®^ punches were transferred into a 96-well, ultra-low attachment plate (Corning, Amsterdam, The Netherlands) and 200 µL D10 medium supplemented with 50 µg/mL l-ascorbic-2-phosphate (AAP) (Sigma-Aldrich, St. Louis, MO, USA, D10-A) was added. The HDFs were harvested and a cell concentration of 0.4 × 10^5^ cells/50 µL was adjusted. The medium was removed from the Matriderm^®^ punches, and 50 µL of the cell suspension was injected into the punches (see Figure 1c). After 15 min incubation at 37 °C 150 µL, D10-A medium was added to the wells. The dermal equivalents (DEs) were cultivated in an incubator with a temperature of 37 °C and a CO_2_ concentration of 5% for 8–11 days, whereby the medium was exchanged three times a week (200 µL D10-A for each well).

**Figure 2:**
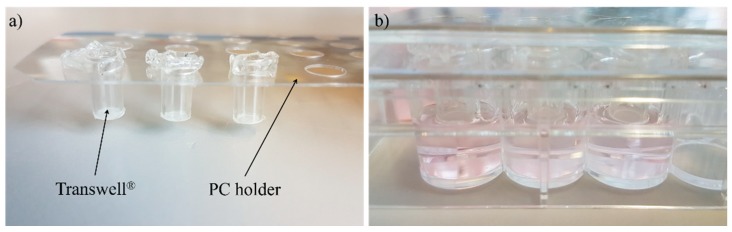
Pictures of the 48-well plate Transwell^®^ holder. (**a**) The Transwell^®^ can fitted in the polycarbonate (PC) holder plate and (**b**) positioned in a 48-well plate.

In the next step, the DEs were transferred into cell culture inserts (Transwells^®^, polycarbonate membrane, 0.4 µm pore size from Corning, Amsterdam, The Netherlands) (see Figure 1d); these were set in a customized polycarbonate holder and placed on a 48-well plate (Corning, Amsterdam, The Netherlands) (see Figure 2). Then, 800 µL D10-A medium was added into the bottom of the well and 75 µL into the cell culture insert on top of the DEs. While a standard 96-well receiver plate is normally used for the culture of reconstructed human epidermis models, ftSEs require a larger volume of medium per square centimeter culture area. Therefore, the described customized polycarbonate holder was developed. The DEs were cultivated further for 3–6 days, and the D10-A medium was changed three times a week.

One day before seeding the keratinocytes, the DEs were sealed to the cell culture insert by the addition of a fibrin gel solution (see Figure 1e). Fibrinogen is immediately polymerized to fibrin if thrombin is added. Therefore, two solutions were prepared and transferred to 500 µL reaction tubes. A quantity of 10 µL per DE of fibrinogen solution (composed of 8 µL HDF (0.4 × 10^5^ cells/8 µL) solution, 1 µL D10, and 1 µL fibrinogen (100 mg/mL, Baxter, Vienna, Austria)) and 10 µL thrombin solution (composed of 8 µL CaCl_2_ (40 mM in H_2_O; Sigma-Aldrich, St. Louis, MO, USA) and 2 µL thrombin (4 U/mL; Baxter, Vienna, Austria)) was needed. The medium on top and below the DE was removed, and 10 µL thrombin solution was mixed with 10 µL fibrinogen solution. The mixture was transferred immediately to the DEs. The clot was incubated for 30–60 min at 37 °C in the CO_2_-incubator for polymerization. The dermis equivalents were cultured one day further in D10-A medium, as described above.

On the next day, NHKs were harvested, and a cell concentration of 0.8 × 10^5^ cells/50 µL was adjusted with the E1 medium supplemented to 1.5 mM CaCl_2_ (E2). The medium was removed from the ftSEs, and 50 µL of the cell suspension was added on top (see Figure 1f). After incubating for 2 h at 37 °C, 15 µL of the E2 medium were added into the top well and 900 µL into the bottom well below the ftSE. After seven days of submerged cultivation (medium changed three times a week) the medium was removed, and the ftSEs were further cultivated in an air lift (ALI) for 10 days (see Figure 1g). For the ALI cultivation, the E3 medium, composed of the E2 medium supplemented with 50 µg/mL AAP and 5 ng/mL keratinocyte growth factor (Sigma-Aldrich, St. Louis, MO, USA), was used (800 µL medium, in the bottom well only), and the medium was changed three times a week.

### 2.3. Cultivation in Two-Organ Chip

**Figure 3:**
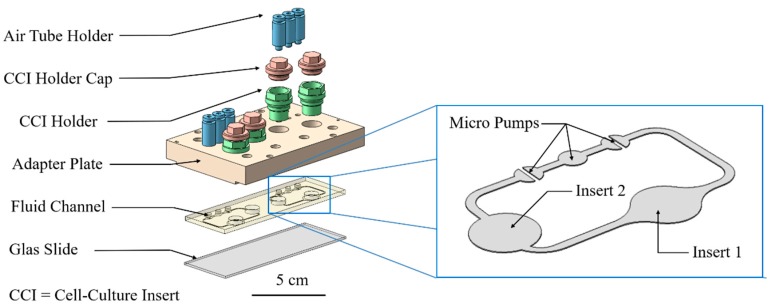
Exploded diagram of the Two-organ chip system. The blue box shows a zoom on the fluid channel with the position of the micro pumps, insert 1 and insert 2.

One part of the ftSEs were cultivated further in a 2OC, produced and provided by TissUse GmbH (Berlin, Germany) (see Figure 3). Further information about the 2OC can be found in Wanger et al. [20], Materne et al. [19], and Schimek et al. [25]. Prior to cultivation, the 2OC was flushed with an E3 medium. The culture compartments of the chip were both filled with 300 µL of the E3 medium. Prior to integration into the chip, the Transwell^®^ containing the ftSE was cut off below the bracket with a heated scalpel and removed. The Transwells^®^ were then positioned air bubble-free into the Transwell^®^-holder, which was screwed into one of the 2OC compartments. The other compartment of each circuit of a 2OC was used as a media reservoir, and was filled up with 600 µL of the E3 medium. The 2OCs were connected to the TissUse Control Units, adjusted to a pump rate of 2 Hz, and incubated at 37 °C and 5% CO_2_. The medium was changed every second day.

### 2.4. Staining

At the end of the culture, the tissues were harvested and frozen in cryomedium TissueTek (Sakura Finetek Germany GmbH, Staufen, Germany) and cut with a Cryotome in slices with a thickness of 8 µm. For the immunofluorescence staining the skin slices were fixed in acetone (Carl Roth, Karlsruhe, Germany) at −20 °C for 10 min, washed with PBS and blocked with 10% goat serum (Sigma-Aldrich, St. Louis, MO, USA) in PBS for 20 min. Tissues were then incubated with the primary antibody over night at 4 °C and washed with PBS. Subsequently, the conjugated secondary antibody and 4,6-diamidine-2-phenylindole (DAPI) (Sigma-Aldrich, St. Louis, MO, USA) (1:2000 diluted in PBS) were added and incubated for 45 min. The sections were washed twice in PBS, covered with mounting medium and stored at −20 °C in the dark until imaging. The antibodies are listed on Table 1.

For the hematoxylin and eosin (HE) staining, the slides were fixed in acetone for 10 min at −20 °C and then washed in distilled water. The slides were then stained in haematoxylin-solution (Harris, VWR, Radnor, PA, USA) for 5 min, and washed again in distilled water. The slices were differentiated by two quick dips in 0.3% acid alcohol. The staining turned blue when tap water was run over the slides for 5 min. Staining in eosin (Merk, Darmstadt, Germany) followed for 2 min, after which the slides were washed again in tap water and then dehydrated in alcohol solutions with increasing concentration (70, 96, and 99% and Roti^®^-Histol (Carl Roth, Karlsruhe, Germany)) for 5 min each. The stained slides were covered with Roti-Histokitt (Carl Roth, Karlsruhe, Germany), and a cover slip was attached.

### 2.5. Permeation Measurement

**Figure 4:**
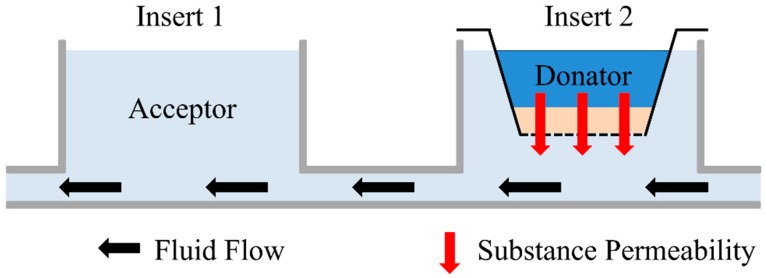
Schematic side view of the 2OC. The acceptor, where the samples are taken, is located at Insert 1. The 96-well CCI system with ftSE is positioned on Insert 2. The donator is the investigated substance on the ftSE. Black arrows indicate the direction of the fluid flow, and the red arrows the permeation.

The permeation experiments were performed in 2OCs. The fluid channels of the chips were filled with E2 medium, and the ftSEs were transferred in the 2OC as described before (see Figure 4). The medium reservoir compartment was used for the acceptor, and was filled with 300 µL E2 medium. Fluorescein sodium salt (Carl Roth, Karlsruhe, Germany) with a concentration of 0.1 mg/mL in E2 medium was used as donator substance, and 75 µL of donator substances were applied onto the top of the ftSE in the CCI. The acceptor (receiver plate, bottom of the ftSE) was filled with 300 µL E2 medium (see Figure 4). During the experiment, the 2OC was positioned in the incubator at 37 °C and 5% CO^2^. Hourly, 100 µL samples were taken from the acceptor and transferred to a 96-well plate to measure the fluorescence. After the measurement, the sample was returned into the 2OC.

To determine the permeation coefficient, the following equation derived from Fick’s first law was used:

(1) $$\frac{dc_{A}}{dt}=P\;\times \;A\frac{c_{D}}{V_{A}},$$

where $c_{A}$ is the concentration of the substance at the acceptor, $c_{D}$ is the concentration at the donator, t is time, P is the permeation coefficient, A is the permeation surface, and $V_{A}$ is the volume of the acceptor. This equation can only be applied when $c_{A}\gg c_{D}.$

### 2.6. Numerical Simulation

The numerical simulation was executed with the program COMSOL Multiphysics (Version 5.1, COMSOL Inc., Stockholm, Sweden) under the following assumptions:

(a) The diffusion coefficient of fluorescein sodium salt in H_2_O was set to a value of 1 × 10^−9^ m^2^/s, which is approximately 100× higher than the diffusion coefficient through the ftSE.
(b) In the experiment, the substance diffuses through the ftSE, and a membrane of the CCI system. This is considered to be one homogenous phase.
(c) For the computation fluid dynamic (CFD) simulation, all physical boundary effects on walls were neglected.
(d) The fluid flow in the 2OC was assumed to be a laminar. The experimentally measured mean value was used as flow velocity.

**Figure 5:**
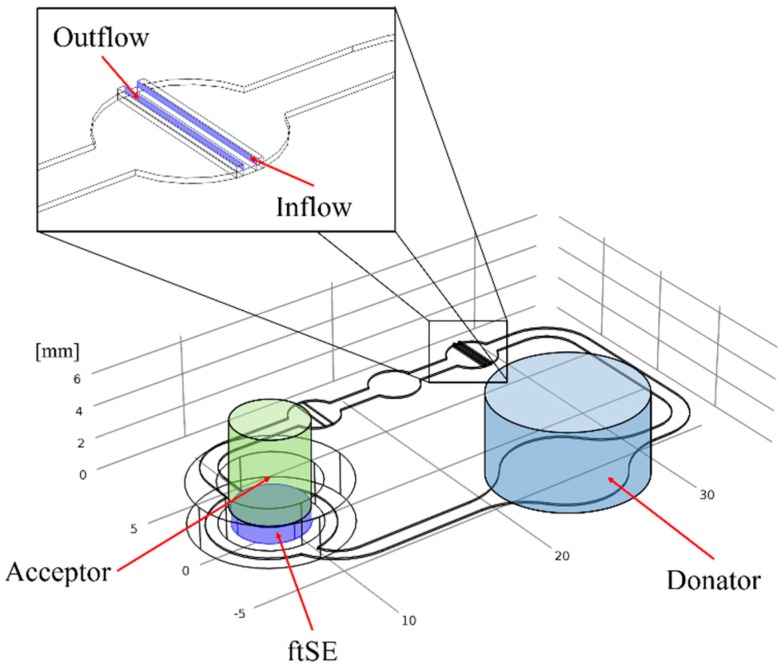
Geometry of the numerical simulation. The green cylinder shows the volume of the acceptor, and the blue cylinder shows the volume of the donator. At the bottom of the acceptor the ftSE is located. The zoom shows the position of the in- and outflow for the fluid simulation.

The fluid channel geometry of the 2OC was implemented in the simulation. On the position of Insert 1, a cylinder was generated with the acceptor volume. The donator and ftSE volume were also realized as a cylinder, and located at the Insert 2 position (see Figure 5). Two small cuboids were positioned on the micro pump for the in- and outflow of the fluid simulation (see Figure 5, zoomed picture). There is a gap between the cuboids that was not traversable for the flow. The volumes correspond to the real experimental setup.

**Figure 6:**
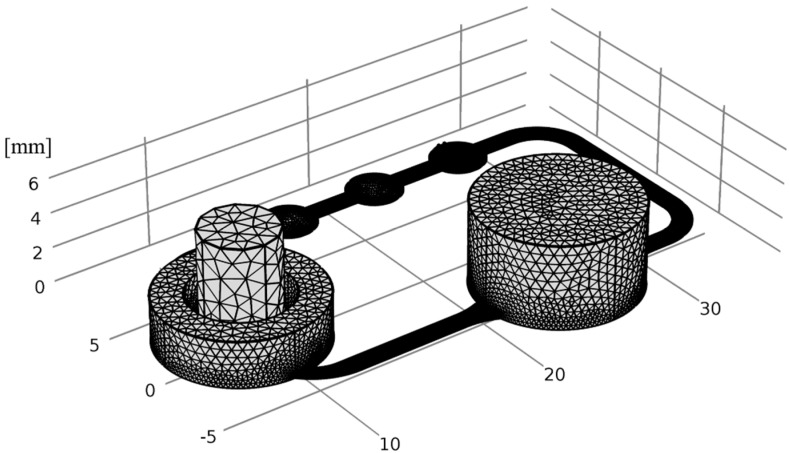
The mash of the numerical simulation. To generate the grit, the auto mash function with “normal” tetrahedral element size was used.

For the fluid flow simulation, the “laminar flow” module was used. All areas affected by the fluid flow were included in the CFD (exceptions are the donator, the ftSE, and the gap). The material in this area was declared as water. The position of the in- and outflow of the fluid were defined on the two small cuboids, mentioned above. The velocity was set at a value of 15.76 mm/s. A stationary study with a GMRES (generalized minimal residual method) solver was used to simulate the laminar flow. The integrated auto mash was used to generate the grid, and the element size “normal” was selected (see Figure 6).

The diffusion process of fluorescein sodium salt was simulated with the “transport of diluted species” module. All areas except for the ftSE were assigned a diffusion value of 1 × 10^−9^ m^2^/s. The diffusion coefficient of the ftSE was assigned a value (calculated from the parameter adaption). All areas circulated by the liquid have been coupled to the CFD. A time-dependent study with a nonlinear Newton’s method was used to simulate the diffusion process. The time range was set from 0 s to 20,000 s, with a time step of 100 s.

The diffusion coefficient of the ftSE was determined with the “optimization” module. For this, the experimental data were integrated in the “global last-square objective”. The first column of the data was defined as the time, and the second column expresses the concentration of the donator. The concentration of the donator was set as the average concentration of the cylinder on Insert 1, and the “global control variable” was set with an initial value of 1 and a control range between 0–1000. For the parameter adaption, the global control variable was multiplied by the factor 1 × 10^−10^. The diffusion coefficient of ftSEs was set as the chosen global control variable, in order to optimize the value at this area. The study of this module was implemented in the time-dependent study of the “transport od diluted species”, and the SNOPT (Spares Nonlinear Optimizer) method with tolerance of 1 × 10^−8^ was chosen. Exact step-by-step instructions for the simulation on an CCI system with the similar simulation modules can be found in Hsu et al. [22].

## 3. Results

### 3.1. Histological Analysis of the Full-Thickness Skin Equivalents

**Figure 7:**
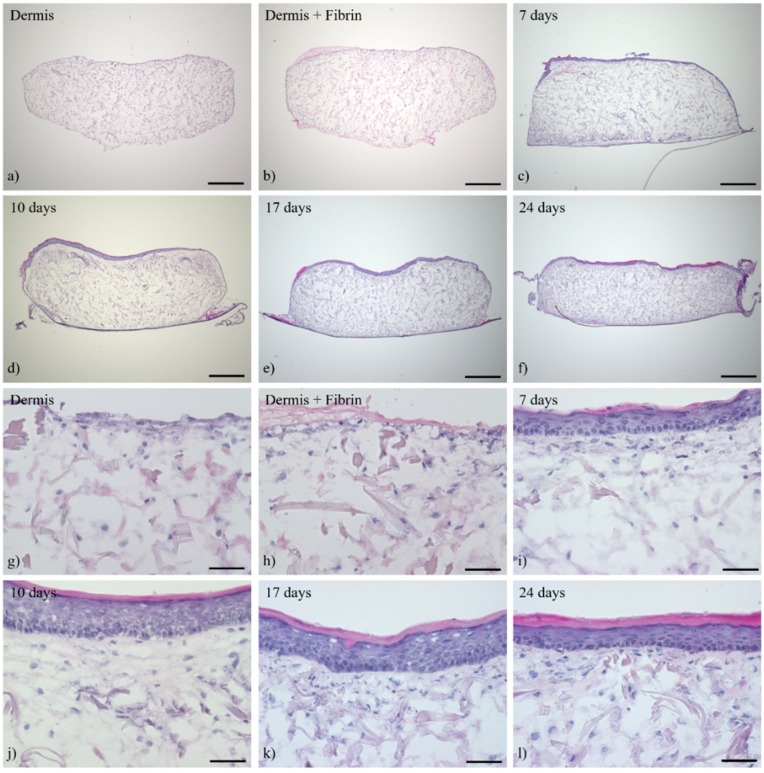
Hematoxylin and eosin (HE) staining of full-thickness skin equivalents (ftSEs). The ftSE morphology was evaluated at different time points. Images show representative cuts of ftSEs of (**a**,**g**) dermis only, (**b**,**h**) dermis with fibrin gel added on top, and (**c**,**i**) after seven days, (**d**,**j**) 10 days, (**e**,**k**) 17 days, and (**f**,**l**) 24 days of ALI culture. The skin equivalents developed a multilayered and stratified epithelium during this 24-day period. (**a**–**f**) Scale bar: 500 µm. (**g**–**l**) Scale bar: 50 µm.

The cutaneous tissue morphology and expression pattern of several differentiation markers have been analyzed, using histological and immunohistochemical staining at different time points over a culture period of 24 days in the ALI, in order to characterize ftSEs over a long-term culture period. Histological staining shows that HDFs are distributed homogeneously throughout the dermis matrix after 14 days pre-culture (Figure 7a,g). A thin layer of fibrin glue sealed the gap between the colonized matrix and the CCI wall. As a result, an accumulation of fibroblasts formed at the upper part of the dermal equivalent (Figure 7b,h). Several epidermal layers were already apparent after seven days of ALI cultivation (Figure 7c,i). An increase in the number of layers was visible until day 10. Furthermore, a thicker *stratum corneum* and an epithelium containing more keratohyalin granules in the *stratum granulosum* emphasized the advanced differentiation process (Figure 7d,j). No differences between 10 and 17 days of ALI culture could be recognized, except for further thickening of the stratum corneum (Figure 7e,k). After 24 days, all layers were still present, but seemed to be more compact (Figure 7f,l). Keratinocytes of the basal layer were regularly arranged with a columnar structure throughout the cultivation period (Figure 7i–l).

**Figure 8:**
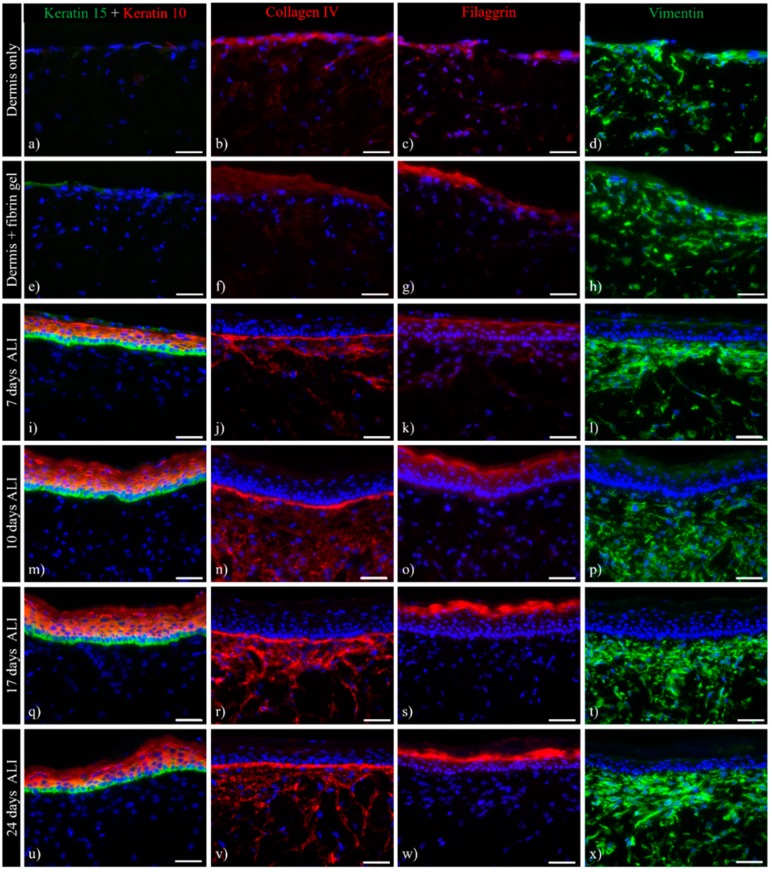
Immunohistochemistry of ftSEs. Expression of skin-specific markers was observed over time. Representative images of (**a**,**d**) dermis only and (**e**,**f**) dermis with added fibrin gel, as well as ftSEs after (**i**–**l**) seven days air lift (ALI), (**m**–**p**) 10 days ALI, (**q**–**t**) 17 days ALI, and (**u**–**x**) 24 days ALI were selected for further analysis. Column 1 (**a**,**e**,**i**,**m**,**q**,**u**) shows keratin 10 (red)/15 (green) double immunostaining. Column 2 (**b**,**f**,**j**,**n**,**r**,**v**) shows collagen IV, Column 3 (**c**,**g**,**k**,**o**,**s**,**w**) shows filaggrin, and Column 4 (**d**,**h**,**l**,**p**,**t**,**x**) shows vimentin staining. An increase in epidermal differentiation and basement membrane marker expressions at different time points indicates a physiologically relevant skin development. Counterstaining of cell nuclei with DAPI (blue). Scale bar: 50 µm.

Immunofluorescence analysis should provide an insight into the expression kinetics of the skin equivalents. Differentiation-specific keratin expression patterns are shown here by K10/K15 double staining (Figure 8a,e,i,m,q,u). Without any keratinocytes seeded on top of the dermis, no K10/K15 expression was observed (Figure 8a,e). At all other time points, a strict border between K15-positive basal keratinocytes and K10-positive suprabasal cells was visible (Figure 8i,m,q,u).

Filaggrin protein expression was seen during the epidermal differentiation process. This late differentiation marker is expressed and processed by cells in the stratum granulosum. Filaggrin expression in skin equivalents increased significantly over time (Figure 8c,g,k,o,s,w). While barely visible until seven days of air-lift culture, intense staining was seen after 17 and 24 days of culture, indicating advanced differentiation levels.

The formation of a basal membrane was proven by a prominent collagen IV staining (Figure 8b,f,j,n,r,v). Collagen IV expression within the dermis and basal zone, already present after seven days of ALI culture, increased slightly over time. Deposition of collagen IV was observed, particularly near the dermal–epidermal junction, while decreasing towards the lower dermis (Figure 8r,v).

Vimentin was used as a marker for fibroblasts populating the dermis, which were found to be spread throughout the entire construct (Figure 8d,h,l,p,t,x). An increased number of cells accumulated within the upper part of the dermis construct. This trend continued further when keratinocytes were added on top of the dermis, resulting in a densely-populated upper dermis and a less-densely-populated lower dermis. This phenomenon can also be observed within the papillary and reticular dermis of native human skin in vivo.

**Figure 9:**
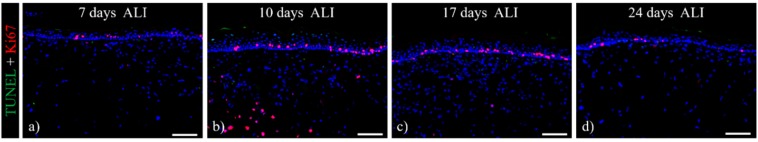
TUNEL-Ki67 stain of ftSEs. TUNEL-positive apoptotic cells are stained in green, and proliferative Ki67 positive cells are stained in red. A considerable number of proliferating cells within the basal layer of the ftSEs and a few apoptotic cells are found after (**a**) 7, (**b**) 10, (**c**) 17 and (**d**) 24 days of ALI cultivation. Counterstaining of cell nuclei with DAPI (blue). Scale bar: 100 µm.

The vitality of the skin equivalents after long-term culture was determined by TUNEL-Ki67 immuno-labelling (Figure 9). The mitotic activity of the keratinocytes, visualized by Ki67 staining, showed a still-considerable number of proliferating keratinocytes within the basal layer after 24 days of air-lift culture. Concurrently, only a few apoptotic TUNEL-positive cells were seen within the ftSE, indicating a still viable skin equivalent. The long-term maintenance and regular differentiation characteristics suggested a balance between proliferation, differentiation, and apoptosis, leading to a physiological tissue homeostasis.

### 3.2. Multi-Organ-Chip Culture

Subsequently, the ftSEs were cultured for seven days in the 2OC to make them suitable for future co-culturing experiments with other organ equivalents.

**Figure 10:**
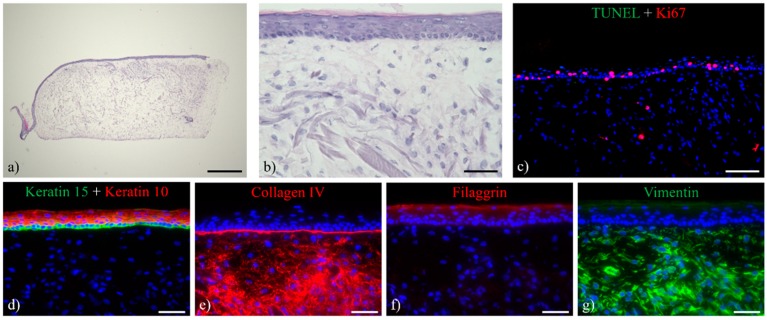
HE staining and immuno-labeling of ftSE cultured for seven days in the 2OC. (**a**,**b**) HE stain of the ftSE showed preserved morphology comprising a multilayered epidermis. (**c**) TUNEL-Ki67 (green and red) staining of the ftSE revealed a considerable number of proliferating cells within the basal layer of the epidermis, while only a few apoptotic cells could be detected. (**d**) Keratin 10 (red)/15 double stain, (**e**) collagen IV (red), (**f**) filaggrin (red), and (**g**) vimentin (green) staining indicate advanced levels of differentiation within the ftSE cultured in the 2OC. Counterstaining of cell nuclei with DAPI (blue). (**a**) Scale bar: 500 µm. (**b**,**d**–**g**) Scale bar: 50 µm. (**c**) Scale bar: 100 µm.

Histological analysis of ftSEs cultivated in the 2OC showed a comparable morphology of ftSEs cultured at static conditions (Figure 10a,b).

Immunohistochemical analysis demonstrates the expression of early differentiation markers (Figure 10d) and the presence of a basement membrane (Figure 10e). The late differentiation marker filaggrin, however, is expressed only weakly (Figure 10f). The high expression of vimentin (Figure 10g) and collagen IV (Figure 10e) within the dermal matrix may reflect a higher proliferative and active level of the residing fibroblasts. The skin equivalents remained viable and proliferative, as indicated by TUNEL/Ki67 staining (Figure 10c).

### 3.3. Trans-Epithelial Electrical Resistance and Permeation

**Figure 11:**
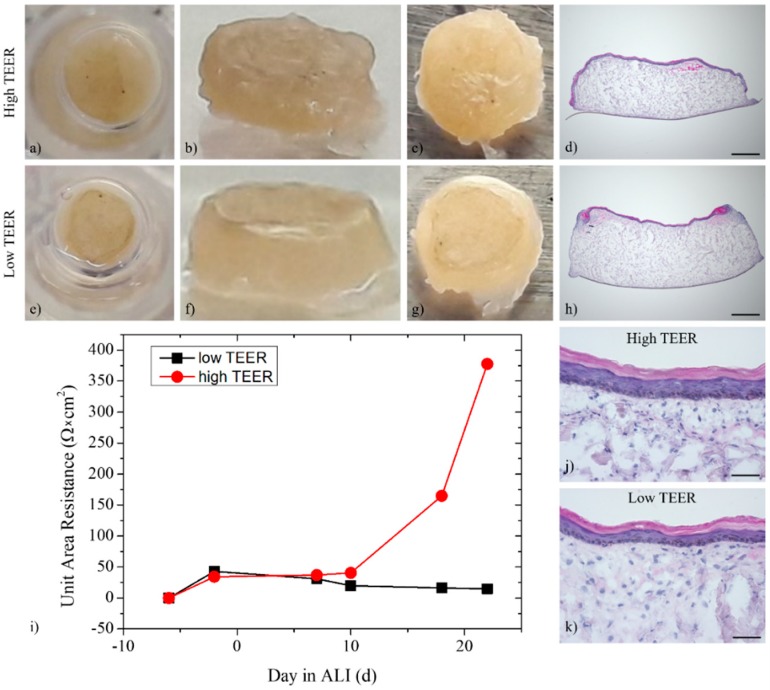
Macroscopic view and HE staining of the ftSE (**a**–**d**,**j**) with high and (**e**–**h**,**k**) low trans-epithelial electrical resistance (TEER). About 40% of the ftSE showed severe contraction. (**i**) Performance of the skin barrier was additionally evaluated by TEER. (**d**,**h**) Scale bar: 500 µm. (**j**,**k**) Scale bar: 50 µm.

**Figure 12:**
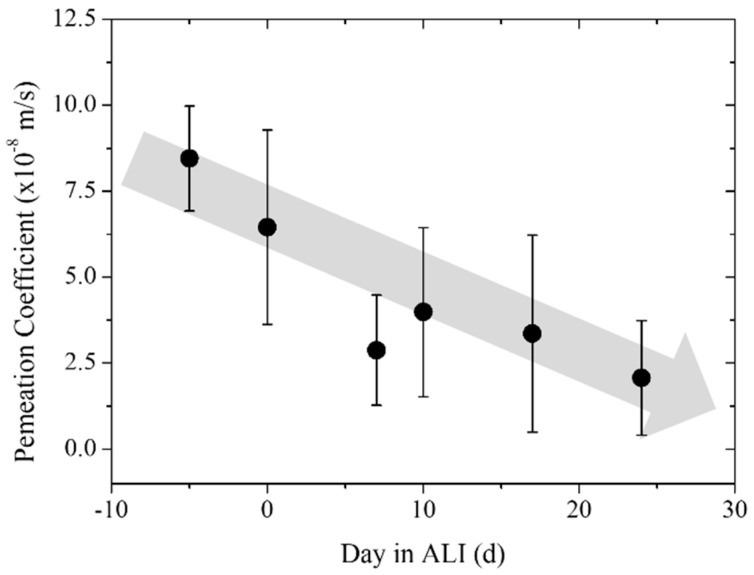
Permeation coefficient over the time of the ftSE. The permeation of fluorescein sodium salt through the ftSEs was observed over time. The ALI was executed at day 0. The days before pre-culture are declared with a minus. A continuous decrease of permeation over time can be seen (gray arrow).

As skin integrity is important for substance permeation tests, the skin equivalents were evaluated macroscopically as well as by measuring the trans-epithelial electrical resistance (TEER) (Figure 11i) and permeation (Figure 12) during the entire cultivation period.

Differences in size and skin integrity could be observed macroscopically. About 40% of the skin equivalents prepared within each experiment failed due to severe shrinkage, overfloating, or other reasons. However, most of the skin equivalents remained stable until the end of the culture. The differences visible macroscopically were confirmed by differing TEER. While skin equivalents with high TEER showed a homogeneous epidermis until the CCI wall (Figure 11a–c), overfloating medium at the edges and the epidermis restricted to the center of the CCI were noticed for skin equivalents with low TEER (Figure 11e–g). This observation could be verified by HE staining (Figure 11d,h). Fewer epidermal layers could have also caused low TEER (Figure 11j,k).

Macroscopically defective skin equivalents were sorted out for the next experiment. The permeation of fluorescein sodium salt through the ftSEs was studied to determine a correlation with corneal differentiation. A permeation coefficient of 8.23 × 10^−8^ m/s was detected during the pre-culture (5 days before ALI). The parameter decreased to a value of 6.45 × 10^−8^ m/s on the day of ALI. After seven days of cultivation in ALI conditions, the coefficient was still decreasing down to a value of 2.88 × 10^−8^ m/s, and increased slightly to a value of 3.99 × 10^−8^ m/s on day 10 of ALI. As the experiment progressed, a permeation coefficient of 3.36 × 10^−8^ m/s was measured on day 17 in ALI, and a coefficient of 2.07 × 10^−8^ m/s was measured at the end, after 24 days of cultivation. Overall, a continuous decrease of the permeation coefficient of fluorescein sodium salt through the ftSEs over the cultivation period was observed (Figure 12). This indicates a relationship between the permeation coefficient and corneal differentiation of the ftSEs. The permeation coefficients, errors, and number of samples are listed in Table 2.

### 3.4. Numerical Simulation

The diffusion coefficient through the ftSE into the 2OC system was evaluated by mathematical approximation. Therefore, a numerical simulation was used to fit the diffusion coefficient with the data from the permeation experiment, described previously.

**Figure 13:**
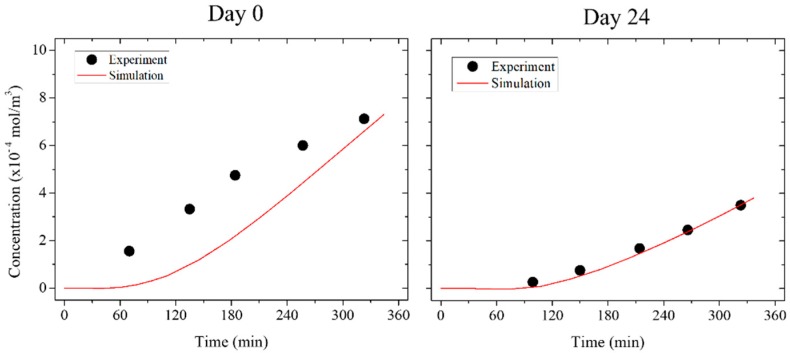
Comparison of the simulation with the permeation experiment. The diagrams show the donator concentration over time. The permeation of fluorescein sodium salt through the ftSE on day 0 (day of the ALI) and day 24 were determinant. The diffusion was calculated with numerical simulation by parameter adaption with Fick’s law.

A diffusion coefficient of 20.45 × 10^−11^ m^2^/s was calculated for the ftSEs in the pre-culture. This value decreased to a value of 4.41 × 10^−11^ m^2^/s on day 0 (day of the ALI). After one week of cultivation, the diffusion coefficient was 2.55 × 10^−11^ m^2^/s, and increased to a value of 2.88 × 10^−11^ m^2^/s on day 10. The diffusion coefficient decreased further to a value of 1.65 × 10^−11^ m^2^/s on day 24 (Table 3). Similar to the permeation experiment, inversely proportional behavior was seen for the ftSEs. Validation of the simulation with the experimental data was achieved by calculating the coefficient of determination (*r*^2^) (Table 3). The values for the different time points are listed in Table 3. The value was highest on day 24, declining with decreasing cultivation time and reaching a value of 0.34 on day 0. The value even fell below zero for the pre-culture. This indicates that the simulation on day 24 matches the experimental data closely, but moves away from the experimental value as it approaches pre-culture. The negative value of *r*^2^ suggests that the mean value adjusts the experimental data better than the simulation. The relationship between the simulation and the experiment is visualized by plotting the data (Figure 13). The simulation shows the same course for the experiment on day 24. At day 0, the concentration of the experimental data increased more rapidly compared to the simulation.

## 4. Discussion

The aim of this work was to develop an ftSE with the size of a 96-well CCI that can be used for an organ-on-a-chip system. These skin equivalents are small, so fewer cells and less substance are needed. Therefore, the ftSE can be used for high throughput assays.

The immunological staining showed regular epidermal development and expression of typical marker proteins. The keratinocytes alter their keratin expression with an increasing differentiation status. Undifferentiated, mitotically-active keratinocytes of the basal layer express the keratins K5/K14 [31] and K15 [32]. When differentiation is initiated in the keratinocytes of the suprabasal layers, expression of K1 and K10 is characteristic [32]. Filaggrin, a protein of the cornified envelope used as a late differentiation marker [33], was found at later stages of ALI. The presence of this protein indicates a regular epidermal development of the ftSEs. Physiological paracrine signaling between keratinocytes in the epidermis and fibroblasts in the dermis is necessary for the development of the basement membrane zone. The crosstalk triggers the synthesis of a complex network of extracellular matrix located at the dermal–epidermal junction. The main components of the basement membrane zone are collagen IV, collagen VII, perlecan, nidogen, and various laminins [34]. The expression of the basement membrane protein collagen IV at the interface of the epidermis and the dermal matrix of the ftSEs provides evidence for their potential to organize themselves in a physiological way. Vimentin is the major intermediate filament protein of mesenchymal cells, and is used widely as a marker for dermal fibroblasts [35]. Interestingly, more fibroblasts accumulated within the upper part of the dermis. This is close to the native dermal architecture, where the fibroblasts are denser in the upper papillary dermis than in the lower reticular dermis [36]. This increased number of fibroblasts could also explain the enhanced collagen IV accumulation in the upper part of the dermis. A leak-proof connection of the skin equivalent with the CCI wall is indispensable for a controlled experiment [37]. The combination of the Matriderm^®^ matrix with a fibrin gel to engineer the dermal component of the ftSE has been proven to be advantageous. The fibrin acts as a sealant, and furthermore, improves keratinocyte growth, as shown in several clinical studies [38,39,40].

According to OECD test guidelines (e.g., No. 428), the integrity of the skin barrier must be evaluated before a substance can be applied for penetration/absorption studies. Transepithelial/transendothelial electrical resistance is a widely accepted quantitative and noninvasive method based on measuring ohmic resistance [33]. As the intention of the ftSEs was to develop skin equivalents compatible with skin penetration/absorption studies, TEER measurements were conducted to verify the ftSEs’ barrier integrity. The TEER recapitulated the findings from macroscopic and histological analyses. Shrinkage and worse epidermal performance both resulted in low TEER values. However, higher TEER was still low in comparison to the TEER desired described in literature. The standard limit value based on experiments with punched human skin samples, for example, was 1 kΩ. However, these values have to be regarded with care, and vary a lot between the laboratories, as the resistance measured is dependent on the device, the frequency applied, the resulting current, the ionic strength of the solution, and the surface area of the skin sample [41]. While TEER measurements are commonly used for RHE characterization [42], they have not been described as routine tools for ftSEs, due to severe shrinkage. One alternative is measuring the impedance across a wide spectrum of frequencies [43], or the permeation measurement of a model substance, as shown here.

Microfluidic technologies have made it possible to control the microenvironment dynamically and allow for the crosstalk of multiple organ systems, by which more reliable drug discovery studies can be conducted [44]. The aim of this study was, therefore, to include the established 96-well CCI-based skin equivalents into the 2OC, as shown previously [18,19,20]. Skin equivalents cultured for seven days within the 2OC remained viable, and showed a nearly consistent epidermal and dermal structure compared to static culture. Collagen IV and vimentin expression within the dermis increased substantially, indicating highly active fibroblasts, most likely due to the dynamic environment constantly replenishing the medium. This is in accordance with findings in other publications describing the culture of ftSEs in organ-on-a-chip systems. For example, EpidermFT™ skin equivalents (MatTek corporation, Ashland, MA, USA) cultured within the 2OC showed rearrangement and compression of the dermal matrix in dynamic cultures and a denser appearance of cell nuclei, indicating an enhanced proliferation of the fibroblasts in dynamic compared to static cultures. However, hardly any difference in viability between statically and dynamically cultured ftSEs was visible after nine days of culture, although viability was slightly worse in static cultures [18]. Research published by Abaci et al. [14] showed long-term maintenance of the dermo-epidermal ftSEs cultured on-chip that was also similar to the static culture. Contrarily, ex-vivo skin biopsies cultured within the 2OC showed substantial differences to the static culture, and the viability and morphology could be preserved over 14 days in a dynamic culture [18]. This indicates the need for perfused culture systems if skin models with increased complexity are used—for example, with integrated hair, vasculature, the immune system, or adipose tissue.

The permeation of substances was measured in a 96-well CCI system in previous experiments [22]. The permeation measurement was transferred to the 2OC system for further applications. The barrier function of the skin is one of its main characteristics. Thus, its permeability can be used as a quality factor to characterize the skin equivalent. It could be shown that the development of the ftSE can be followed with the help of the permeation measurement. It must generally be claimed that the barrier of the ftSE still differs from human skin. Permeation experiments with testosterone [8], aldosterone, hydrocortisone [45], and corticosterone [46], which have a similar molecular size as fluorescein sodium salt, show a permeation coefficient through human skin in the range of 0.4 × 10^−10^ up to 3.76 × 10^−9^ m/s. These are more than ten times smaller than the values measured for the ftSEs. However, this method of permeation determination seems to be promising for assessing the condition of the skin equivalent during the culture. This could represent a possible additional quality control for the production of the skin equivalent.

The simulation is representative of the ftSEs at the last stage of the cultivation, where the skin equivalent is well-differentiated. At the beginning of the culture, the deviation between the simulation and the experiment is high. The actual diffusion process was faster than the simulation. In all likelihood, the diffusion cannot be precisely described with Fick’s law. A possible reason for this effect is the fluid flow of the 2OC. The accelerated distribution of the substance can cause anomalous diffusion [47]. The simulation has to be extended with an equation for abnormal diffusion [48] to account for this effect.

## 5. Conclusions

In conclusion, skin equivalents were generated successfully inside 96-well CCIs, and their implementation into the 2OC proved its suitability as a pharmacological test system for substance penetration studies. Furthermore, it was possible to extend the simulation to the 2OC, and the diffusion coefficient could be determined for differentiated ftSEs. The numerical simulation can be used further to predict long-term effects and support and reduce experimental efforts.

## Figures and Tables

**Table 1:** List of antibodies used for immunofluorescence staining.

Antibodies	Dilution in PBS	Manufacturer
Collagen I, mouse anti-human, 5.9 mg/mL	1:100	Sigma ^1^
Cytokeratin 10, mouse anti-human, 1 mg/mL	1:100	Chemicon ^2^
Cytokeratin 15, rabbit anti-human, 100 µg/mL	1:100	Chemicon ^2^
Filaggrin, mouse anti-human, 0.2 mg/mL	1:50	Thermo Fisher ^3^
IgG–CF 488, goat anti-rabbit, 2 mg/mL	1:200	Biotium ^4^
IgG–CF 594, goat anti-mouse, 2 mg/mL	1:200	Biotium ^4^
Vimentin, IgG rabbit anti-human, 200 µg/mL	1:100	Santa Cruz ^5^

^1^ Sigma-Aldrich, St. Louis, MO, USA. ^2^ Merck, Darmstadt, Germany. ^3^ Thermo Fisher, Schwerte, Germany. ^4^ Biotium, Fremont, CA, USA. ^5^ Santa Cruz Biotechnology, Dallas, TX, USA.

**Table 2:** Result of the permeation experiments with fluorescein sodium salt through the ftSE. The permeation at different times during the cultivation was investigated.

Time (Day in ALI)	Permeation Coefficient (×10^−8^ m/s)	Error (×10^−8^ m/s)	Number of Samples (N)
Pre-culture	8.23	1.53	3
Day 0	6.45	2.83	4
Day 7	2.88	1.60	5
Day 10	3.99	2.46	5
Day 17	3.36	2.86	5
Day 24	2.07	1.66	5
Blank	0.03	0.02	5

**Table 3:** Results of the calculated diffusion coefficient with the numerical simulation and the coefficient of determination (*r*^2^) regarding the experimental data. The diffusion of fluorescein sodium salt through the ftSEs at different cultivation times was investigated.

Time	Diffusion Coefficient (×10^−11^ m^2^/s)	*r*^2^
Preculter	20.45	−19.15
Day 0	4.41	0.32
Day 7	2.55	<0.01
Day 10	2.88	0.49
Day 17	2.66	0.83
Day 24	1.65	0.98
Blank	0.03	0.02
